# Highly Textured Zinc Deposition: A Pathway to Long‐Life Rechargeable Aqueous Batteries

**DOI:** 10.1002/cssc.202502204

**Published:** 2026-03-07

**Authors:** Ang Li, Xinyu Zhang, Maochun Wu

**Affiliations:** ^1^ Department of Mechanical Engineering The Hong Kong Polytechnic University Hong Kong SAR China

**Keywords:** oriented deposition, rechargeable aqueous zn battery, side reactions, Zn dendrites

## Abstract

Rechargeable aqueous Zn batteries (RAZBs) offer compelling advantages for large‐scale energy storage, including intrinsic safety, low cost, and environmental sustainability. Yet, their widespread deployment is hindered by uncontrolled dendrite growth and parasitic side reactions associated with Zn electrodes. Manipulating directional Zn deposition has emerged as one of the most promising strategies to address these challenges. In this perspective, we critically examine recent advances in controlling the orientation of Zn deposition and highlight key mechanisms underpinning directional growth. More importantly, we outline future research priorities to achieve highly textured Zn depostion: unraveling the micromechanisms of oriented deposition, establishing unified evaluation standards for texture, expanding the focus beyond the conventional Zn(002) plane to alternative crystallographic textures, designing deposition strategies resilient to diverse operating conditions, correlating orientation control with full‐cell electrochemical performance, and developing scalable, application‐driven deposition techniques. By integrating theoretical insights with practical considerations, this article aims to chart a path toward high‐performance, commercially viable RAZBs for next‐generation energy storage.

## Graphical abstract

This perspective summarizes emerging strategies for oriented Zn deposition to suppress dendrites for high‐performance rechargeable aqueous Zn batteries and points out future research directions.

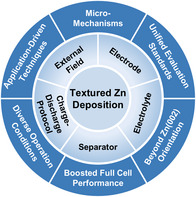

## 1 Introduction

Driven by the imperatives of climate change and the global energy transition, the demand for sustainable, affordable, and secure energy storage solutions is rapidly growing. The surging demand is steering the exploration of electrochemical energy storage technologies beyond lithium‐ion batteries for large‐scale applications [1, 2, 3, 4]. Among various technologies, rechargeable aqueous Zn batteries (RAZBs) have emerged as one of the most promising candidates for this application, owing to the Zn metal anodes with high theoretical capacity (820 mA h g^−1^ or 5855 mA h cm^−3^), low redox potential (−0.76 V vs. standard hydrogen electrode), abundant natural reserves, and inherently safe aqueous electrolytes [5, 6, 7, 8].

Despite their great promise, the widespread commercialization of RAZBs is severely hindered by the poor reversibility and stability of Zn metal anodes during repeated plating/stripping [9, 10]. This limitation primarily arises from uncontrolled Zn dendrite growth and parasitic side reactions [11]. Typically, Zn metal foil is widely employed as the anode material in RAZBs due to its well‐established fabrication process and low cost [12]. Nevertheless, the grains in commercially rolled Zn foil are randomly oriented [13, 14]. Various crystal planes, such as Zn(002), Zn(100), and Zn(101), are randomly exposed on its surface [15, 16]. As Zn nucleation and growth are highly dependent on the crystal structure of the substrate, a phenomenon known as ‘epitaxial deposition’ [17, 18], the nonoriented Zn foil results in disordered Zn deposition [19, 20]. Moreover, commercial Zn foils suffer from surface roughness [21], and their surface is typically covered with naturally formed oxide layers with poor conductivity [22, 23]. These factors contribute to uneven distribution of electric field and Zn^2+^ ion concentration during charging process, further exacerbating the risk of irregular deposition [24, 25]. Even worse, the randomly formed microscopic protrusions will intensify the local electric field, attracting more Zn^2+^ ions to deposit and thus resulting in rampant Zn dendrite growth [19, 26]. The dendritic electrodeposits are porous and exhibit poor adhesion to the substrate [27, 28]. During cycling, these dendrites may fracture and detach from the substrate, forming ‘dead Zn’ that no longer participates in subsequent electrochemical reactions, leading to rapid capacity decay and shortened cycle life [29, 30]. Additionally, the large surface area of dendrites exacerbates side reactions between the metal electrode and aqueous electrolyte [31, 32]. The resulting by‐products such as alkaline zinc sulfate (Zn_4_(OH)_6_SO_4_·*x*H_2_O, ZHS) and zinc oxide further amplify dendrite growth, which may penetrate the separator between electrodes, ultimately resulting in short circuits and thus failure of RAZBs [33].

In recent years, directing the oriented Zn deposition has been increasingly recognized as a crucial strategy to address the aforementioned challenges [34]. By controlling the selective growth of Zn along specific crystallographic orientations, it is possible to achieve highly textured and densely packed deposits with a smooth and uniform macroscopic morphology [35]. This approach fundamentally suppresses dendrite formation and growth, thereby dramatically improving the cycling stability and reversibility of Zn anodes [36]. To date, extensive and fruitful research has been conducted across multiple dimensions, including electrode engineering, electrolyte formulation, separator design, optimization of charge–discharge protocols, and the application of external fields, to promote directional Zn deposition. In this perspective, we first systematically summarize the latest advances in this field, highlighting innovative strategies for guiding oriented Zn deposition, along with their underlying mechanisms and unique advantages. More importantly, we critically assess the limitations of current research and identify key opportunities for future development. Challenges such as insufficient understanding of micro‐mechanisms, inconsistent evaluation criteria, and lack of strategies tailored for real‐world applications are discussed in depth. This perspective aims to provide valuable insights and forward‐looking guidance for the rational regulation of oriented Zn deposition, paving a pathway toward high‐performance RAZBs for next‐generation energy storage.

## 2 Emerging Strategies for Oriented Zn Deposition

### 2.1 Electrode Engineering

**FIGURE 1:**
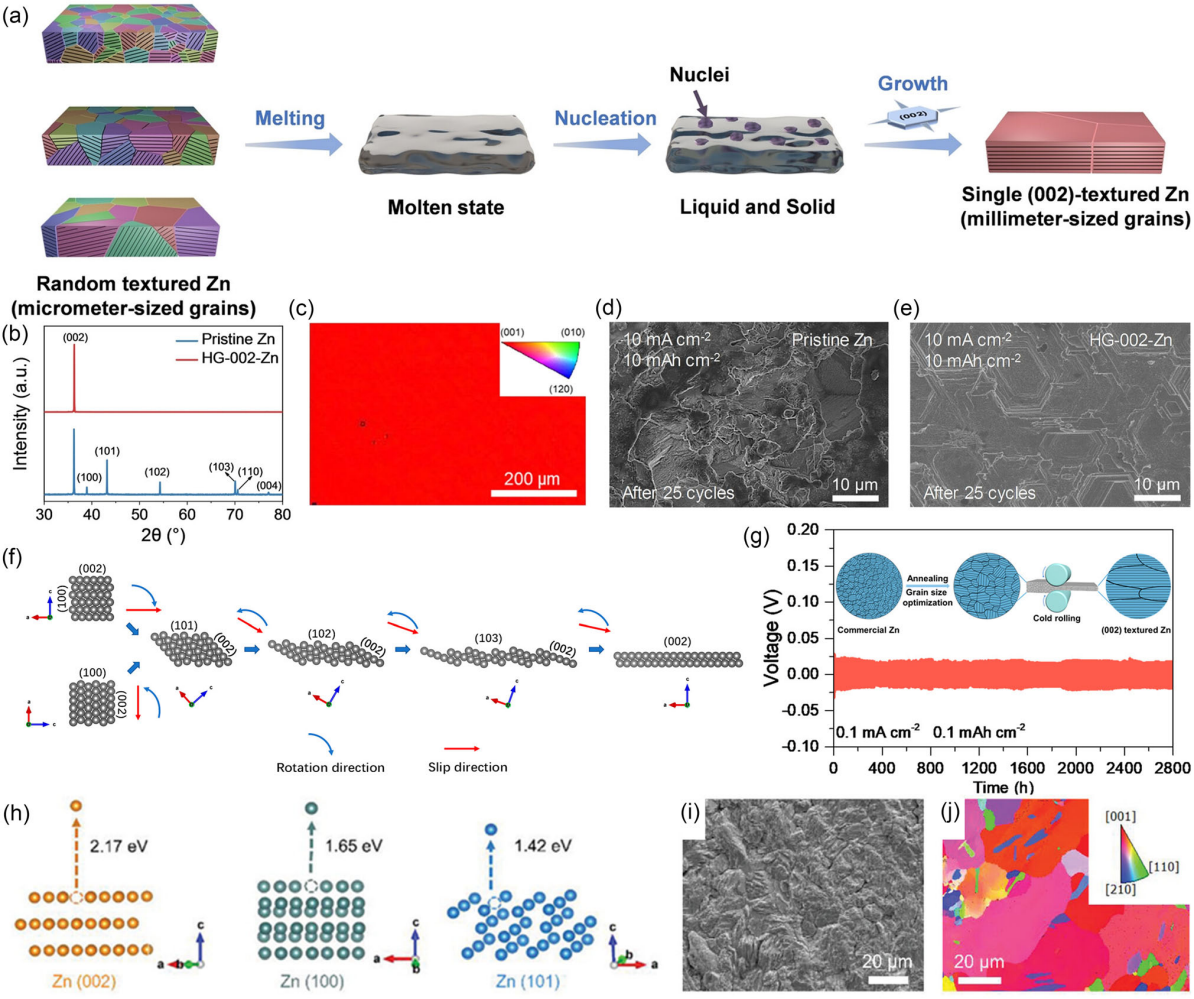
(a) Schematic illustration of the melting‐solidification process for obtaining single (002)‐textured Zn foil. (b) XRD patterns of pristine Zn foil and modified (002)‐textured Zn foil. (c) EBSD map of (002)‐textured Zn foil. Scanning electron microscope (SEM) images of (d) pristine Zn and (e) (002)‐textured Zn foil after 25 cycles at 10 mA cm^–2^ with an areal capacity of 10 mA h cm^–2^. Reproduced from Ref. [40] with the permission from John Wiley and Sons. (f) Schematic illustration of the Zn facets evolution process during cold rolling. (g) Long‐term galvanostatic charge–discharge (GCD) performance of Zn//Zn symmetric battery using designed Zn foil at 0.1 mA cm^–2^ and 0.1 mA h cm^–2^, with insert showing the schematic cold‐rolling. Reproduced from Ref. [41] with the permission from American Chemical Society. (h) *E*_s_ of Zn from different crystal planes (Zn(002), Zn(100), and Zn(101)) obtained from DFT calculations. (i) SEM image and (j) EBSD orientation map of acid‐etched Zn. Reproduced from Ref. [42] with the permission from John Wiley and Sons.

As metallic Zn is electrodeposited onto the electrode, its surface conditions (e.g., crystal structure and chemical composition) play a decisive role in governing the electrodeposition behavior [37]. While commercial Zn foils are widely used as electrodes, the lack of specific crystalline textures and the presence of numerous surface defects often lead to disordered Zn deposition and dendrite growth [38]. To address this issue, various mechanical or chemical pretreatment methods have been developed to engineer specific textures on Zn foils, thereby guiding epitaxial Zn deposition. Liu and coworkers developed an annealed Zn foil through thermal treatment [39]. The heat‐induced recrystallization and nucleation yielded highly (101)‐oriented Zn foil. The engineered texture facilitated epitaxial Zn deposition with minimal lattice distortion, thereby significantly enhancing the cycling stability and extending the battery lifespan. Moreover, Chen et al. rapidly transformed commercial Zn foil into (002)‐textured foil by a melting‐solidification process as shown in Figure 1a [40]. During the melting process, the initial texture and residual stresses of the original foil were eliminated. Upon subsequent solidification, Zn(002) texture spontaneously formed due to its lowest surface energy. This preferential orientation was confirmed by X‐ray diffraction (XRD) patterns (Figure 1b) and electron backscatter diffraction (EBSD) map (Figure 1c). Consequently, the single (002)‐oriented Zn anode enabled epitaxial Zn deposition and demonstrated excellent dendrite suppression capability (Figure 1d,e). In addition, Ma's group reshaped commercial Zn foil into strongly (002)‐textured foil by combining annealing and roll‐pressing processes [41]. First, annealing moderately increased the grain size of Zn foil, enhancing its plastic deformability. The following cold rolling induced a strong (002) crystallographic texture, as illustrated in Figure 1f. When used in Zn//Zn symmetric cells, the modified foil enabled a long cycle lifespan of 2800 h at 0.1 mA cm^−2^ (Figure 1g). Furthermore, Zhang's group employed acid etching to obtain textured Zn foil [42]. Density functional theory (DFT) calculations revealed distinct stripping energies (*E*_s_) for various Zn crystal planes, as shown in Figure 1h, with the Zn(002) plane displaying the highest *E*_s_. This characteristic enables distinct etching rates of different facets in acidic environments: thermodynamically unstable planes (Zn(100) and Zn(101)) were first dissolved by boric acid solution, while the Zn(002) plane remained intact and exposed (Figure 1i,j). The resulting Zn foil enriched with Zn(002) orientation enabled a Zn//Zn symmetric cell to achieve excellent cycling stability (360 h at 20 mA cm^−2^, 20 mA h cm^−2^). A similar approach was adopted by Guo et al. [43]. They utilized electrochemical stripping to selectively expose Zn(002) by taking advantage of the difference in *E*_s_, thereby obtaining a modified Zn foil with enhanced performance.

**FIGURE 2:**
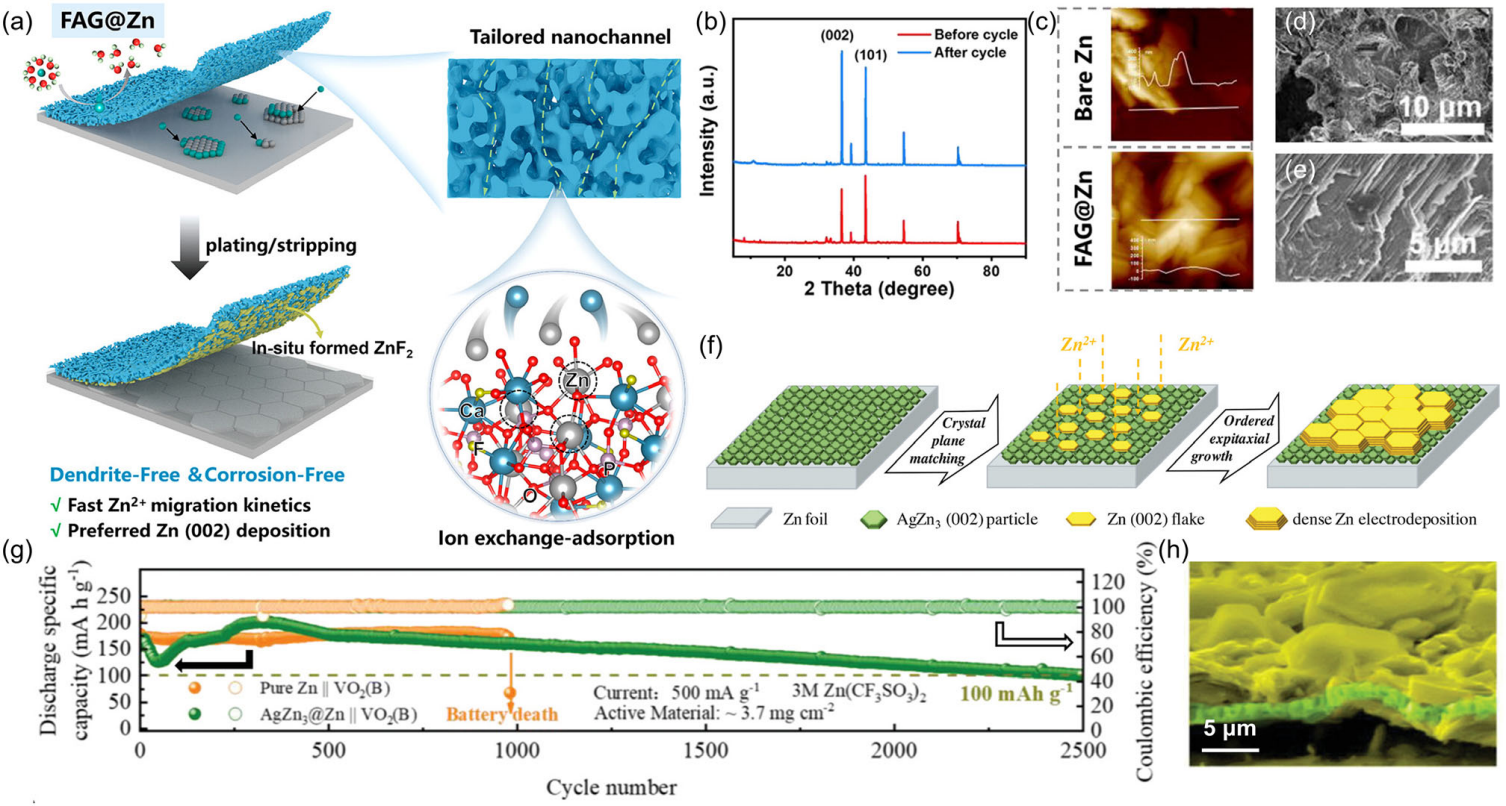
(a) Schematic illustration of FAG‐regulated Zn deposition process. (b) XRD patterns of the FAG@Zn anode before and after cycling. (c) Atomic force microscope (AFM) images of the different anodes after cycling, with the insert showing the surface profile. SEM images of (d) blank Zn and (e) FAG@Zn anodes after cycling. Reproduced from Ref. [50] with the permission from American Chemical Society. (f) Schematic illustration AgZn_3_ alloy layer‐induced epitaxial (002) orientation deposition. (g) Long‐term GCD performance of VO_2_(B) full cells with pure Zn and AgZn_3_@Zn anodes. (h) The pseudo‐color SEM image of Zn deposits formed on AgZn_3_@Zn anode. Reproduced from Ref. [51] with the permission from John Wiley and Sons.

Designing artificial interfacial layers is another promising strategy to guide textured Zn deposition. Based on their electronic conductivity and Zn deposition sites, these interfacial layers can be categorized as artificial protective layers and initial Zn nucleation layers. The artificial protective layer permits ionic transport while suppressing electronic conduction, thereby directing Zn^2+^ deposition beneath the coating layer. An ideal design should satisfy the following criteria. First, the protective layer should display chemical and structural stability in the electrolyte and accommodate repeated volume changes of the Zn anode during plating/stripping cycles. Second, it must exhibit sufficient uniformity and compactness to effectively regulate Zn^2+^ transport. Moreover, the protective layer should feature high Zn^2+^ conductivity and low electron conductivity. Finally, the protective layer must demonstrate strong adhesion to the metal substrate. A wide range of protective layer materials has been reported, including inorganic materials (carbon‐based materials, metal oxides, metal nitrides, metal selenides, and inorganic salts), organic materials (polymers, organic acids, amino acids, metal–organic frameworks, and covalent organic frameworks), and their composites [44, 45, 46, 47, 48]. Consequently, a variety of preparation techniques, including the squeegee method, spin coating, solvent casting, wet‐chemical methods, and chemical vapor deposition, have been developed [49]. For instance, Shi et al. coated a fluorapatite (Ca_5_(PO_4_)_3_F) aerogel (FAG) protective layer onto the Zn surface [50]. This coating layer provided abundant Zn adsorption sites within uniformly distributed nanoscale channels as shown in Figure 2a. It effectively homogenized and accelerated Zn^2+^ ion transport, promoting uniform nucleation on the anode surface, and thus (002)‐oriented, dendrite‐free Zn deposition was achieved (Figure 2b–e). The designed FAG coating enabled a Zn//Zn symmetric battery to stably operate for over 2000 cycles at 1 mA cm^−2^ and 0.5 mA h cm^−2^. Furthermore, Shu et al. fabricated an artificial cerium oxide (CeO_2_) layer on the Zn anode [52]. The CeO_2_ facilitated the horizontal growth of Zn along the (002) crystal plane, effectively suppressing dendrite formation and enabling symmetric cells to achieve stable charge–discharge cycling for 520 h at a current density of 10 mA cm^−2^ and an areal capacity of 10 mA h cm^−2^.

In contrast to Zn deposition underneath the protective layer, the initial nucleation layer guides ordered Zn deposition on its surface. Unlike protective layers, initial nucleation layers should exhibit high electronic conductivity and limited ionic conductivity to ensure electron transport through the layer while constraining Zn deposition to its surface. Additionally, the nucleation layer should possess high zincophilicity and/or a low lattice mismatch with metallic Zn to facilitate epitaxial growth. Two‐dimensional materials such as hexagonal boron nitride and VSe_2_ have demonstrated the ability to direct Zn deposition via lattice matching mechanism [53, 54]. A more widely adopted strategy for forming an initial nucleation layer involves engineering Zn alloy anodes through chemical replacement reactions or physical deposition techniques [55]. For instance, Yang's group successfully fabricated an AgZn_3_ alloy layer through magnetron sputtering [51]. This alloy demonstrates strong zincophilicity and features a (002) texture that closely matches the Zn(002) crystal plane, thereby enabling dendrite‐free, (002)‐directional Zn deposition, as illustrated in Figure 2f. As a result of this controlled nucleation behavior, the AgZn_3_@Zn//VO_2_(B) full cell maintained stable operation for up to 2500 h at 0.5 A g^−1^, far exceeding the cell based on pristine Zn foil (Figure 2g,h). These results underscore the critical role of tailored alloy nucleation layers in achieving uniform Zn deposition and long‐term cycling stability.

**FIGURE 3:**
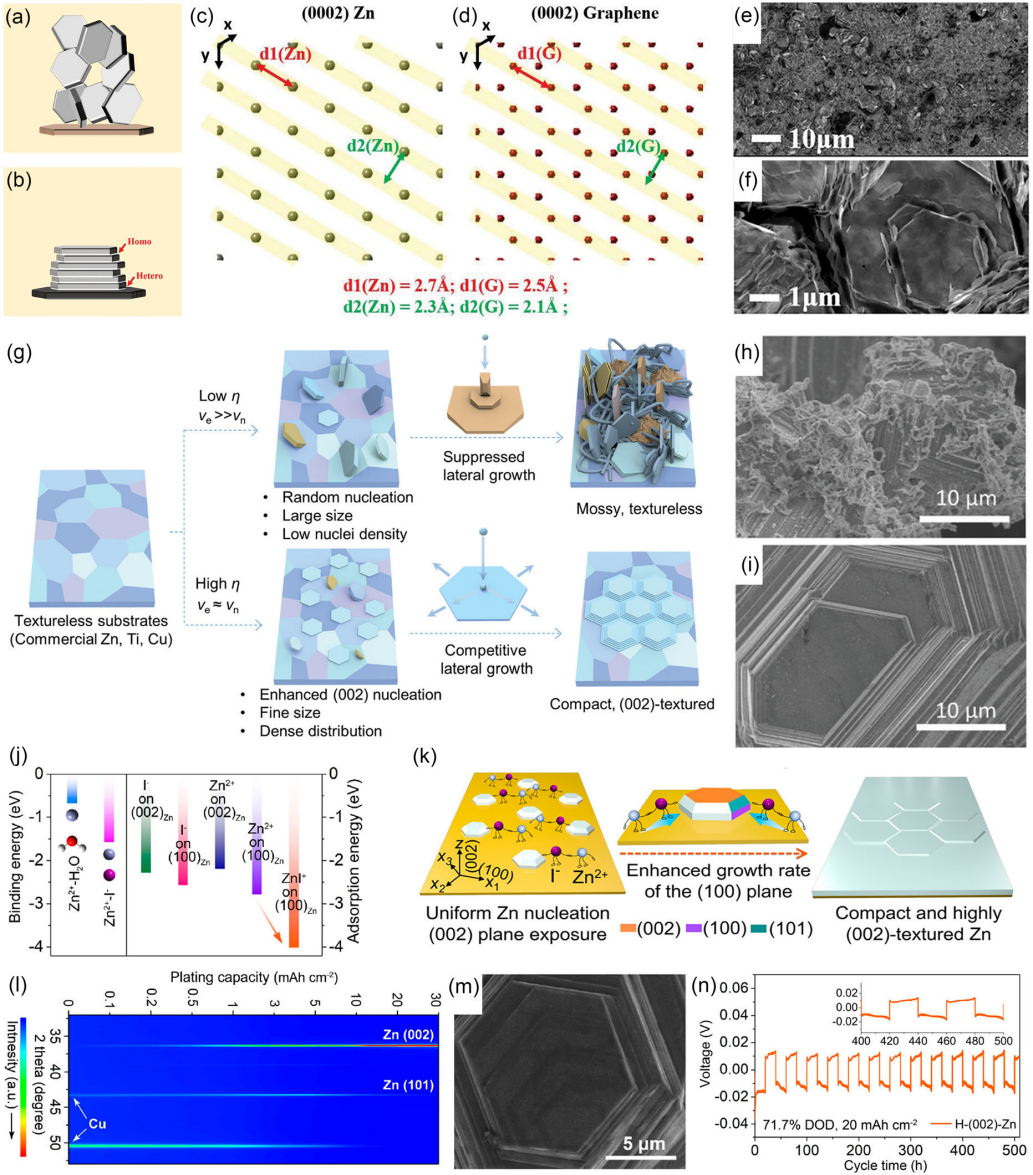
(a,b) Schematic illustration of the epitaxial deposition mechanism. Atomic arrangements of (c) Zn (0002) and (d) graphene (0002) crystal planes. (e,f) SEM images of Zn electrodeposits on graphene‐coated stainless steel at 4 mA cm^−2^ for 12 min. Reproduced from Ref. [56] with the permission from American Association for the Advancement of Science. (g) Schematic illustration of the Zn deposition process on textureless substrates with low and high current densities. SEM images of Zn deposits at (h) 10 mA cm^−2^ and (i) 100 mA cm^−2^. Reproduced from Ref. [35] with the permission from John Wiley and Sons. (j) Binding energies and adsorption energies obtained from DFT calculations. (k) Schematic illustration of the I^–^ ion‐assisted ordered Zn deposition. (l) In situ XRD characterization of Zn deposits in I^–^‐added electrolyte. (m) SEM image of Zn deposit on the Cu substrate in I^–^‐added electrolyte. (n) Long‐term GCD performance of symmetric battery using the designed (002)‐oriented Zn anode at 1 mA cm^–2^ and 20 mA h cm^–2^. Reproduced from Ref. [57] with the permission from American Chemical Society.

In addition to modifying commercial Zn foils, novel strategies for fabricating Zn metal electrodes continue to emerge. Among these, electrolytic deposition stands out as a versatile and scalable route for fabricating high‐quality Zn electrodes. By precisely tuning electroplating parameters, including substrate type, current density, applied potential, and electrolyte composition, it is feasible to achieve the deterministic control over crystallographic texture of Zn foils. In a landmark study from 2019, Archer's group first introduced a (002)‐oriented graphene layer on stainless‐steel substrates [56]. With a lattice mismatch of as low as 7% with the Zn(002) crystal plane (Figure 3c), the graphene interface induced heterogeneous epitaxial Zn deposition and mitigated lattice strain (Figure 3a,b). Figure 3e,f clearly show that the morphology of the resulting Zn deposits is plate‐like rather than dendritic, enabling a Zn//MnO_2_ full cell to achieve outstanding stability over 1000 cycles. Pu's group and Zhang's group provide further insights into current‐dependent Zn deposition behavior [35, 58]. They found that low current densities promote epitaxial Zn nucleation on nontextured substrates, followed by constrained lateral growth along the Zn(002) plane (Figure 3g). This pathway ultimately yields moss‐like, randomly oriented Zn deposits (Figure 3h). In contrast, a moderate current density raises the nucleation overpotential and shifts the growth mode from epitaxial to nonepitaxial. This transition favors the formation of horizontally arranged Zn (002) nuclei, which preferentially grow laterally due to the low adsorption energy of Zn(002) plane and a low diffusion barrier for Zn adatoms. Consequently, competitive lateral growth is established (Figure 3i). Leveraging these findings, (002)‐oriented Zn anodes were successfully fabricated via high‐current electrodeposition on untextured substrates, which significantly extended cycling lifetime when used in batteries. To mitigate lattice distortion during electrodeposition, arising from the low adsorption energy of the Zn(002) plane, and to avoid hydrogen embrittlement at high overpotentials, Zou et al. developed (112)‐oriented Zn anodes via constant‐potential electrodeposition at moderate potentials [59]. RAZBs assembled with the newly developed Zn electrodes exhibited outstanding cycling stability and reduced polarizations. Moreover, Yuan et al. introduced I^−^ ions into the electroplating solution and successfully produced highly (002)‐oriented Zn anodes [57]. DFT calculations revealed that I^−^ ions are preferentially adsorbed on the Zn(100) plane, promoting rapid Zn deposition along this plane (Figure 3j). As a result, the (100) crystal plane gradually diminished during electrodeposition as shown in Figure 3k, which is supported by its near absence of characteristic XRD peaks (Figure 3l). The resulting electroplated Zn electrode exhibited dominant (002) orientation and a smooth surface (Figure 3m), enabling a symmetric cell to stably cycle for over 500 h with high depth of discharge (DOD) of 71.7% (Figure 3n). Subsequently, the same research group discovered that organic cations can also induce highly (002)‐oriented, nonepitaxial Zn electrodeposition [60]. Specifically, methylimidazolium ions are selectively adsorbed onto the Zn(002) crystal plane, thereby retarding electrodeposition on this facet. As a result, a large‐area, free‐standing, highly (002)‐oriented Zn anode was prepared via electrodeposition in methylimidazolium‐added electrolyte, enabling a Zn//VOH full cell to operate for up to 4500 cycles at 2 A g^−1^ with a high‐capacity retention of 79%. To further achieve single (002)‐oriented electrodeposited Zn metal, Zhang's group developed a cation‐anion coregulation strategy [61]. By integrating accelerated effect driven by I^−^ ions adsorbed on the (100) plane and inhibited effect mediated by 1‐ethyl‐3‐methylimidazolium cations adsorbed on the (002) plane, they obtained samples exhibiting a relative texture coefficient of 100% for the Zn(002) plane. Consequently, symmetric batteries assembled with the prepared single (002)‐oriented Zn demonstrated remarkable deep cycling stability (200 h at up to 88% DOD).

### 2.2 Electrolyte Formulation

While the primary function of electrolytes is to facilitate ion conduction, their physicochemical properties critically govern the Zn deposition process, including ion transport, desolvation, and reaction kinetics, thereby affecting the crystal texture and morphology of the deposited Zn. Therefore, tremendous efforts have been made to formulate electrolytes that can promote highly textured Zn deposition.

**FIGURE 4:**
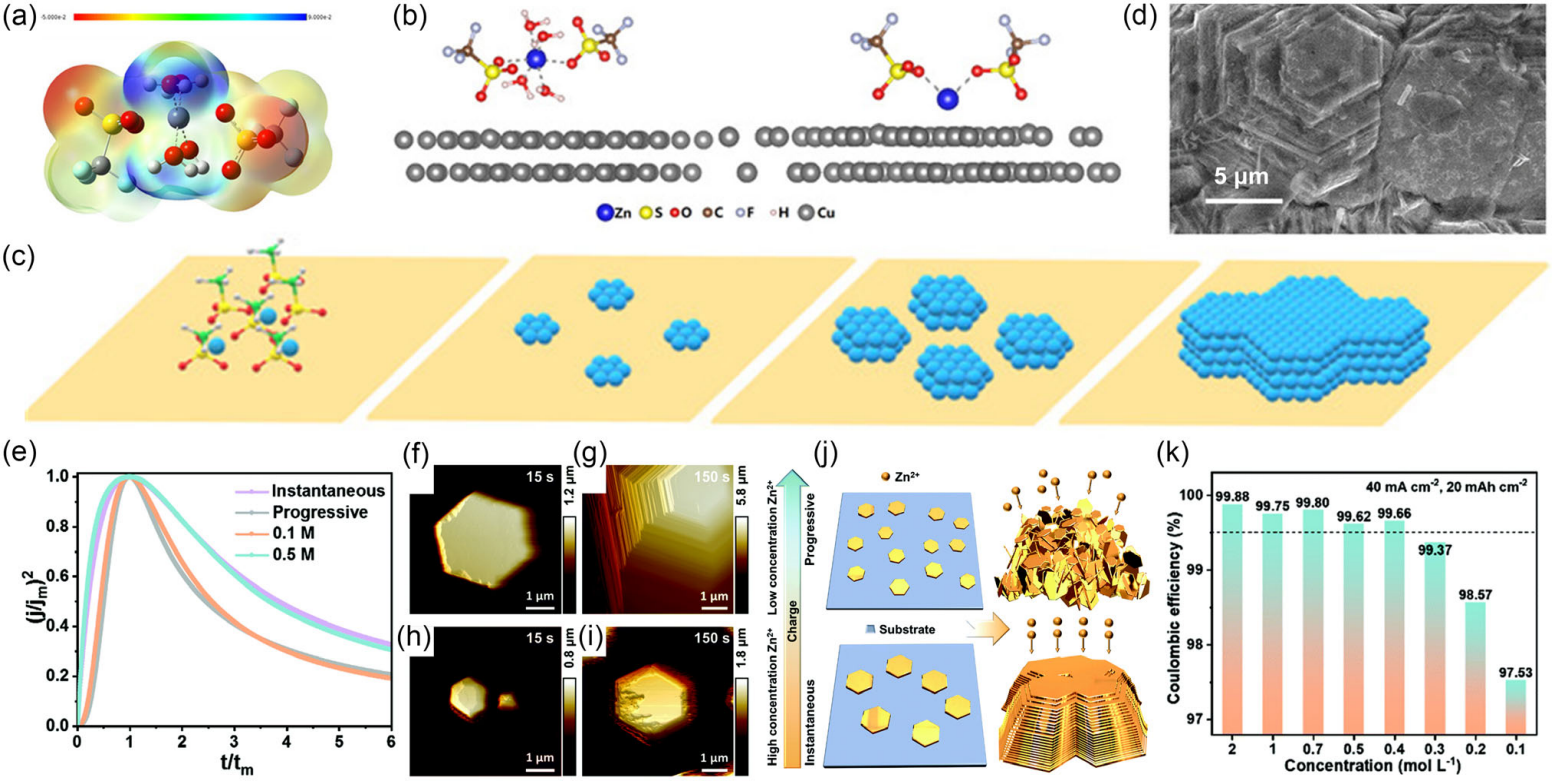
(a) Electrostatic potential distribution of solvated Zn^2+^ in Zn(OTf)_2_ electrolyte obtained from DFT calculations. (b) Optimized structures of solvated Zn^2+^ and Zn(OTf)_2_* on Cu electrode surface. (c) Schematic illustration of the OTf^−^‐induced (002)‐oriented deposition. (d) SEM image of the Zn after cycling in Zn(OTf)_2_ electrolyte at 10 mA cm^−2^ and 10 mA h cm^−2^. Reproduced from Ref. [62] with the permission from John Wiley and Sons. (e) Comparison of dimensionless current–time transients from experimental nucleation at different Zn^2+^ concentrations with theoretical transients for instantaneous and progressive nucleation. AFM images of Zn deposits with a current density of 10 mA cm^−2^ and 0.5 M of Zn^2+^ after (f) 15 s and (g) 150 s. AFM images of Zn deposits with a current density of 10 mA cm^−2^ and 0.1 M of Zn^2+^ after (h) 15 s and (i) 150 s. (j) Schematic illustration of Zn deposition process with different concentrations. (k) Average CEs for 100 cycles (0.1 M for 40 cycles) of graphite plate//Zn asymmetric cells with different Zn^2+^ concentrations at 40 mA cm^−2^ and 20 mA h cm^−2^. Reproduced from Ref. [63] with the permission from Royal Society of Chemistry.

Anions in the electrolyte not only serve as ‘background ions’ to balance charge and maintain conductivity but also play a pivotal role in regulating Zn deposition behavior by modulating ion transport and/or even directly participating in interfacial electrochemical reactions. For instance, Yuan and coworkers demonstrated that trifluoromethanesulfonate (OTf^−^) ions can coordinate with Zn^2+^ ions via their sulfonate groups in Zn(OTf)_2_ electrolyte (Figure 4a) [62]. At the anode‐electrolyte interface, the strong interaction between the oxygen in OTf^−^ and the substrate metal enhances the adsorption of the fully desolvated Zn(OTf)_2_* species as shown in Figure 4b. This promotes the formation of a high density of planar nucleation sites, which evolve into hexagonal flake‐like Zn and eventually coalesce into a metallic film exposing the Zn(002) crystal plane (Figure 4c,d). Moreover, the concentration of Zn salts can modulate deposition texture by influencing nucleation patterns. Li's group investigated the concentration‐dependent morphology and crystal texture of deposited Zn in ZnBr_2_ electrolyte by electrochemical tests and AFM observation [63]. In diluted electrolytes (<0.3 M), progressive nucleation resulted in mossy, nonoriented Zn deposits (Figure 4e,h–j). In contrast, relatively concentrated electrolytes (≥0.4 M) favored instantaneous nucleation, leading to densely packed, blocky deposits with dominant (002) crystal plane exposure (Figure 4e–g,j), which is beneficial for the stability and reversibility of the battery. Notably, an asymmetric cell using a critical concentration (0.4 M) electrolyte exhibited stable cycling for 150 cycles at 40 mA cm^−2^ and 20 mA h cm^−2^ with a high Coulombic efficiency (CE) of 99.66% (Figure 4k).

**FIGURE 5:**
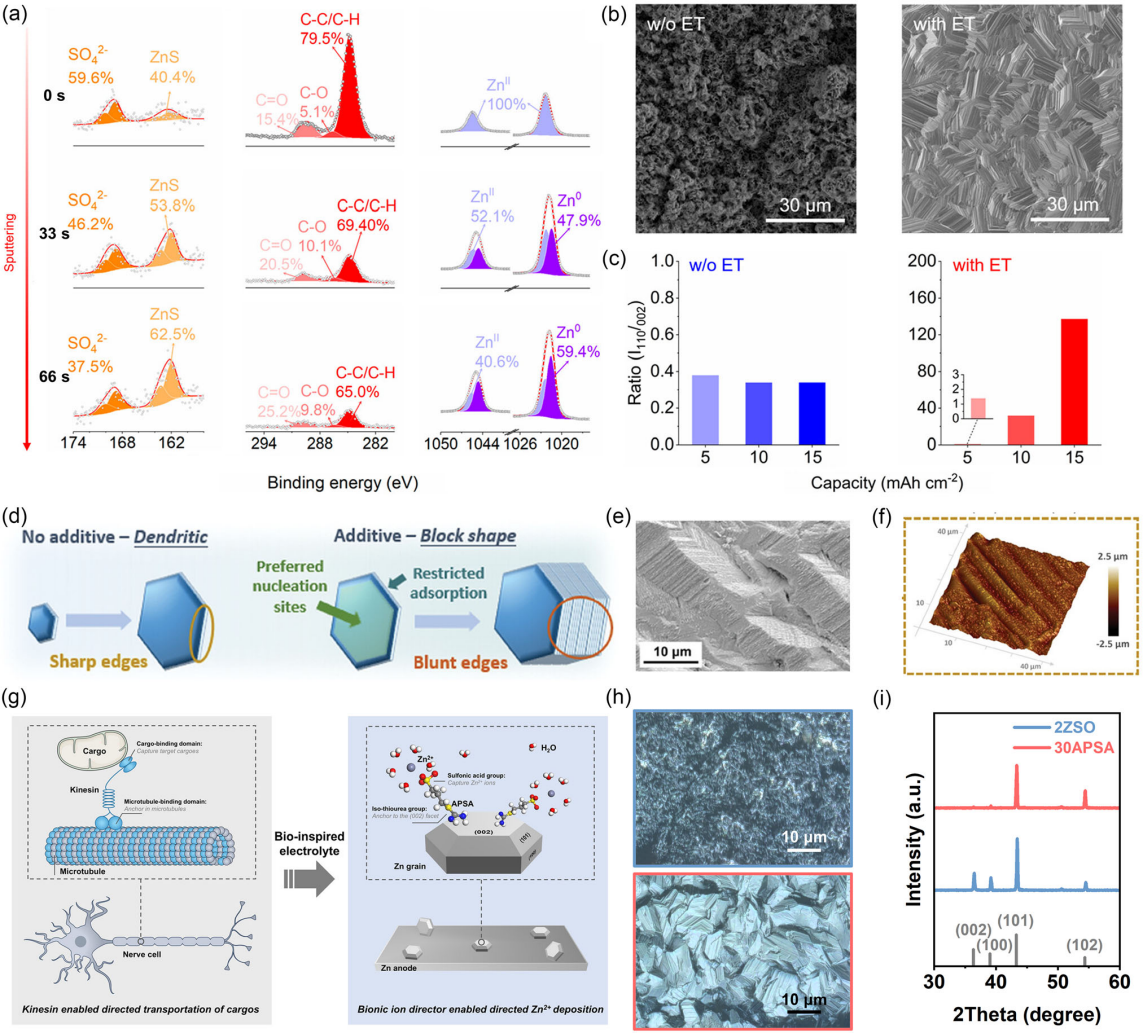
(a) X‐ray photoelectron spectroscopy results of Zn deposits obtained in the ET‐added electrolyte. (b) SEM images of Zn deposits formed in blank and designed electrolytes. (c) Intensity ratio of the characteristic peaks for Zn(110) and Zn(002). Reproduced from Ref. [65] with the permission from American Chemical Society. (d) Schematic illustration of the “edge shielding” mechanism. (e) SEM and (f) AFM images of Zn electrode cycled in Li^+^‐containing electrolyte. Reproduced from Ref. [66] with the permission from John Wiley and Sons. (g) Schematic illustration of the kinesin cargo‐transport process and bionic‐additive‐regulated Zn deposition. (h) Confocal laser scanning microscopy (CLSM) images of Zn deposits at 10 mA cm^−2^ in pristine and APSA‐added electrolytes. (i) Corresponding XRD patterns. Reproduced from Ref. [2] with the permission from John Wiley and Sons.

Introducing electrolyte additives or cosolvents is also a widely adopted strategy to induce oriented deposition in RAZBs. Based on their (electro‐)chemical stability, these additives are generally categorized as either sacrificial or nonsacrificial. Sacrificial additives typically transform into new compounds conducive to Zn deposition at the interface through displacement reactions, electrochemical reduction, or polymerization. For example, Tang et al. developed an In^3+^‐containing electrolyte to in situ build a ZnIn alloy layer on the anode surface [64]. Owing to its high zincophilicity, this alloy layer enhances Zn deposition kinetics and promotes (002)‐oriented Zn deposition. Sun's group introduced erythritol (ET) into a conventional ZnSO_4_ electrolyte [65]. During electrodeposition, a hybrid solid electrolyte interphase containing ZnS and organic components was formed as a result of additive‐mediated decomposition reactions (Figure 5a). The in situ formed interphase induced (110)‐oriented Zn deposition with a uniform surface as shown in Figure 5b,c. Chen and coworkers formulated a ZnSO_4_ and acrylamide‐added electrolyte [67]. During cycling, ZnSO_4_ converted to ZHS while acrylamide underwent in situ polymerization at the interface. The resulting composite protective layer integrates the high mechanical strength of ZHS with the high toughness of polyacrylamide matrix, effectively homogenizing Zn^2+^ ion transport and inducing (002)‐oriented Zn deposition.

In contrast to sacrificial additives, nonsacrificial additives remain stable throughout battery cycling, enabling dynamic and continuous regulation of the directional Zn deposition. Their fundamental mechanism involves selective adsorption or spatial distribution on specific Zn crystal planes during electroplating, thereby modulating the growth rate of targeted facets. For instance, when inert, positively charged cationic additives such as Li^+^ ions or 1‐ethyl‐1‐methylpyrrolidinium cations are introduced into the electrolyte, they migrate under the influence of electric field toward the sharp edges of hexagonal Zn flakes (i.e., Zn(100) and Zn(101) crystal planes) [20, 66]. This effect inhibits Zn growth in these regions with high local electric fields, thereby promoting deposition on the adjacent Zn(002) plane (Figure 5d). Ultimately, this ‘edge shielding’ strategy facilitates the exposure of Zn(101) and Zn(103) crystal planes and results in uniform, dendrite‐free Zn deposits as shown in Figure 5e,f. In addition, the differential adsorption energies of additives on various crystal planes can be exploited to control the deposition orientation. Hu's group reported that disodium maleate (DMA) exhibits a more negative adsorption energy on the Zn(002) plane compared to other facets [68]. Consequently, DMA is preferentially adsorbed onto the Zn(002) plane, redirecting Zn deposition to alternative planes and enabling layered stacking with (002) plane exposure. As a result, a Zn//Zn symmetric battery employing DMA‐containing electrolytes sustained a long cycling lifespan of 120 h at a high current density of 30 mA cm^−2^ and an areal capacity of 30 mA h cm^−2^. Another pathway to achieve oriented growth is to accelerate Zn deposition on targeted facets. Li et al. introduced a kinesin‐mimetic ion director, 3‐(amidinothio)‐1‐propanesulfonic acid (APSA), to realize crystal plane‐selective deposition [2]. The isothiourea groups of APSA selectively adsorb onto Zn(002) facets, like the microtubule‐binding domain of kinesin, while the sulfonic acid groups concentrate Zn^2+^ near the anchored sites, enhancing electrodeposition kinetics, which resemble cargo‐binding domains as illustrated in Figure 5g. This bio‐inspired approach enables dynamic, self‐adaptive regulation of Zn^2+^ ion flux, resulting in dendrite‐free deposition with the dominant exposure of the Zn(101) crystal plane (Figure 5h,i).

In addition, to address leakage issues associated with liquid electrolytes and to meet the requirements of flexible energy storage devices, hydrogel electrolytes have been developed in recent years [69, 70]. However, similar to liquid electrolytes, Zn dendrite formation caused by uneven deposition remains a significant challenge for hydrogel electrolytes. Therefore, manipulating the oriented deposition of Zn is also essential to achieve long‐life Zn anodes in these electrolyte systems. In this regard, Zheng's group designed and synthesized hydrogel electrolytes based on zinc dodecylbenzenesulfonate, acrylamide, and 4‐acryloylmorpholine [71]. Compared to the Zn(100) and Zn(101) crystal planes, dodecylbenzenesulfonate ions are preferentially adsorbed onto the Zn(002) surface. This facet‐dependent adsorption inhibits Zn^2+^ deposition on the (002) plane, ultimately exposing this facet during electrodeposition and thereby inhibiting dendrite growth.

### 2.3 Separator Design

**FIGURE 6:**
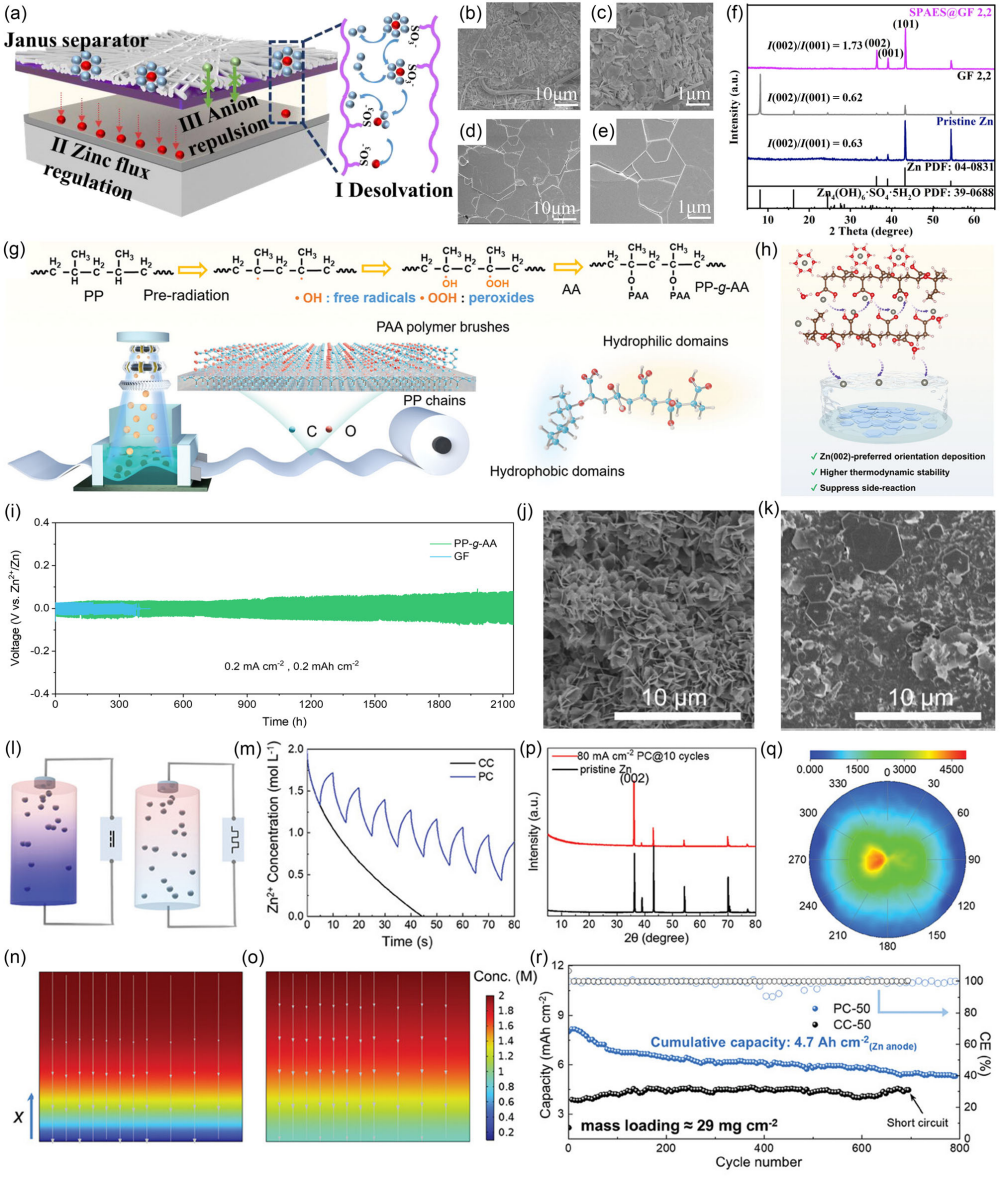
(a) Schematic illustration of the SPAES separator‐regulated Zn deposition. SEM images of Zn anodes after long‐term GCD tests at 2 mA cm^−2^, 2 mA h cm^−2^ with (b,c) GF and (d,e) SPAES@GF separators, and (f) corresponding XRD patterns. Reproduced from Ref. [72] with the permission from Elsevier. (g) Schematic illustration of the preparation process of the PP‐*g*‐AA separator. (h) Schematic illustration of the Zn deposition with PP‐*g*‐AA separator. (i) Long‐term GCD performance of the Zn//Zn symmetric battery using PP‐*g*‐AA and GF separators at 0.2 mA cm^−2^ and an areal capacity of 0.2 mA h cm^−2^. SEM images of Zn anodes after cycling with (j) GF and (k) PP‐*g*‐AA separator. Reproduced from Ref. [73] with the permission from John Wiley and Sons. (l) Schematic illustration of Zn^2+^ concentration distribution under CC and PC protocols. (m) Zn^2+^ concentration‐time profiles at the anode‐electrolyte interface and (n,o) Zn^2+^ concentration distribution after deposition with CC or PC protocols obtained from the numerical simulation (p) XRD patterns of the pristine Zn foil and the Zn after cycling with PC protocol. (q) Pole figure of the cycled Zn. (r) Long‐term GCD performance of Zn//NH_4_V_4_O_10_ full cells with high‐mass‐loading cathodes under PC and CC protocols. Reproduced from Ref. [34] with the permission from John Wiley and Sons.

Separator is another key component in RAZBs, primarily serving to prevent direct contact between positive and negative electrodes while permitting ion transport. In addition to its basic function, a well‐designed separator can regulate Zn^2+^ ion transport and desolvation behavior, thereby inducing directional Zn deposition. For instance, Zhao's group designed a mechanically robust composite separator comprising a sulfonated poly(arylene ether sulfone) (SPAES) dense layer supported by a porous glass fiber (GF) as shown in Figure 6a [72]. The porous GF substrate ensures excellent electrolyte wettability, while the sulfonate‐rich SPAES dense layer promotes the desolvation of hydrated Zn^2+^ ions and guides their uniform migration. This innovative design facilitated horizontal (002)‐oriented deposition (Figure 6b–f), enabling a Zn//Zn symmetric cell to achieve a plating/stripping cycle life exceeding 2000 h. Given the critical role of separators, considerable efforts have been devoted to developing novel separator materials. Zhu and coworkers prepared a hydrophilic separator, PP‐*g*‐AA, by surface grafting acrylic acid onto an electron‐beam irradiated polypropylene substrate (Figure 6g) [73]. The resulting separator exhibited uniform pore size, ultrathin thickness (25 µm), high mechanical strength (tensile strength up to 7.5 MPa), and high ionic conductivity (3.16 mS cm^−1^). The highly polar carboxyl groups on the polyacrylic acid segments homogenized Zn^2+^ ion flux, promoting directional deposition along the Zn(002) plane (Figure 6h). Notably, a Zn//Zn symmetric cell using the PP‐*g*‐AA separator exhibited a cycling lifespan approximately seven times longer than that of a cell using a conventional GF separator at a current density of 0.2 mA cm^−2^ (Figure 6i).

### 2.4 Optimization in Charging and Discharging Protocols

Apart from engineering the above key components, optimizing the charging and discharging protocols has been demonstrated to be an effective approach to inducing directional deposition. In constant‐current (CC) deposition, appropriately increasing the current density promotes (002) texture formation, as described above for the preparation of electrolytic Zn foils. However, within the confined space and limited electrolyte volume of a RAZB, raising current density that exceeds the mass transfer limit leads to Zn^2+^ ion depletion at the interface, exacerbating dendrite growth and side reactions (Figure 6l). To overcome this transport limitation, Zhang et al. proposed a pulsed‐current (PC) protocol [34]. Numerical simulations indicate that intermittent constant‐current deposition avoids interfacial Zn^2+^ ion depletion, as shown in Figure 6m,n. This strategy successfully induced (002)‐textured Zn deposition in a full battery configuration (Figure 6p,q), enabling high‐mass‐loading Zn//NH_4_V_4_O_10_ full cells to achieve long‐term stable charge–discharge performance at high rates (Figure 6r).

### 2.5 Application of External Fields

**FIGURE 7:**
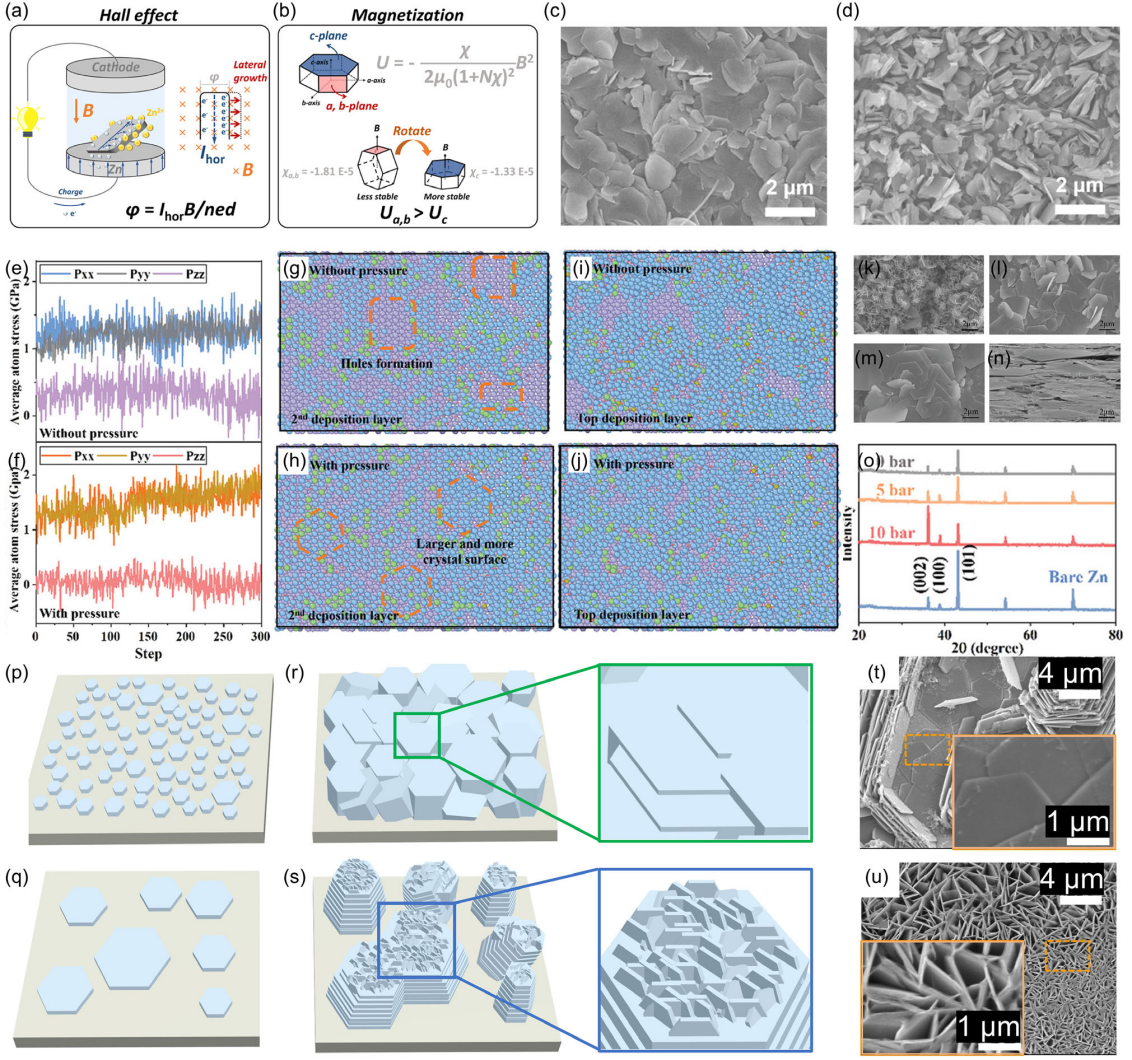
(a) Schematic illustration of motion of electrons driven by the Hall effect. (b) Schematic illustration of the rotation of Zn flake under the magnetic field. SEM images of Zn anodes after 30 cycles at 1 mA cm^−2^ and 1 mA h cm^−2^ (c) with and (d) without a vertical magnetic field. Reproduced from Ref. [74] with the permission from Elsevier. Average surface atomic stress (e) without and (f) under pressure. Top view of the second deposition layer formed (g) without and (h) under pressure. Top view of the final deposition layer formed (i) without and (j) under pressure obtained from MD simulations. SEM images of Zn deposits at a current density of 1 mA cm^−2^ under pressure of (k) 0, (l) 5, (m) 10 bar, and (n) perspective view, and (o) corresponding XRD patterns. Reproduced from Ref. [75] with the permission from John Wiley and Sons. Schematic illustration of the Zn nucleation and growth at (p,r) low temperature and (q,s) high temperature. SEM images of Zn deposits with an areal capacity of 5 mA h cm^−2^ at (t) 25°C and (u) 60°C. Reproduced from Ref. [76] with the permission from Elsevier.

External physical fields, including temperature, pressure, and magnetic fields, can also significantly influence electrodeposition behavior and serve as effective tools for controlling Zn deposition orientation. For example, Zhang et al. achieved Zn(002)‐oriented electrodeposition by applying a magnetic field perpendicular to the electrode surface [74]. This strategy leverages the Hall effect to increase the electron density at the edge of the Zn flakes (Figure 7a), thereby promoting lateral growth. Meanwhile, magnetic anisotropy induces magnetic moments in Zn nuclei, causing them to rotate and align parallel to the substrate as shown in Figure 7b, thereby exposing the (002) crystal plane. Compared to the disordered deposits in the control group (Figure 7d), the application of a magnetic field yielded uniform deposits composed of horizontally aligned Zn flakes (Figure 7c). Luo's group employed reactive molecular dynamics (MD) simulations to investigate the influence of pressure on Zn electrodeposition, gaining atomic‐scale insights into Zn deposit formation at the interface [75]. Their results revealed that applied pressure reduces atomic stress along the pressure direction while increasing stress perpendicular to it (Figure 7e,f), thereby promoting Zn atom diffusion in the perpendicular direction as shown in Figure 7g–j. Guided by these findings, Zn was electrodeposited under standard and external pressure. SEM and XRD characterizations confirmed that pressure promotes the formation of densely packed Zn(002) planes parallel to the substrate (Figure 7k–o). Temperature‐dependent deposition behavior was explored by Li et al. [76]. They studied the evolution of Zn crystal under varying thermal conditions and found that at relatively low temperatures, numerous fine grains grew parallel to the substrate, ultimately forming (002)‐oriented deposits as shown in Figure 7p,r,t. As the temperature increased, enhanced deposition kinetics reduced nucleation density, resulting in large grains and vertical growth of Zn flakes (Figure 7q,s,u).

## 3 Concluding Remarks and Perspectives

In summary, this article presents a critical review of recent advances in directing oriented Zn deposition, which represents one of the most promising strategies to tackle the persistent challenges of dendrite formation and side reactions on Zn electrodes. A wide variety of approaches, ranging from modifying key components (electrode, electrolyte, and separator) to fine‐tuning operational parameters (e.g., current density, temperature, and pressure), have been demonstrated to be effective in promoting preferential Zn nucleation and growth. This directional control enables the formation of flat, compact, and dendrite‐free Zn morphologies, thereby markedly improving the electrochemical performance and long‐term stability of RAZBs. Despite these remarkable advancements, several critical limitations remain unresolved. The following critical directions are suggested to warrant urgent investigation in the future.

### 3.1 Unraveling the Micromechanisms of Oriented Deposition

A deeper understanding of the fundamental mechanisms governing oriented Zn deposition is essential. Future research should integrate in situ and operando characterization techniques with high temporal and spatial resolution to monitor the evolution of Zn crystal planes, morphological changes, and interfacial environments in real time during electroplating and stripping processes [5]. While theoretical modeling has been employed to gain atomic insights into the early nucleation process, current DFT calculations often rely on vacuum or implicit solvent models, which fail to accurately capture interfacial adsorption behavior in realistic electrolyte environments [2, 77]. Future efforts should be made to develop more accurate and multiscale computational models to clarify the thermodynamic and kinetic fundamentals of oriented deposition. Integrating artificial intelligence techniques, such as machine learning, could facilitate the construction of multiscale theoretical models spanning from atomic migration to macroscopic deposition [78, 79, 80]. This would provide theoretical guidance for deposition control and reduce reliance on empirical trial‐and‐error approaches.

### 3.2 Establishing Unified Evaluation Standards for Texture

Future efforts should establish unified standards for characterizing oriented Zn deposition behavior, including quantitative methods for determining orientation degree, grain size, and orientation uniformity, to facilitate comparison and validation across different research outcomes. Currently, commonly used techniques such as SEM and XRD provide direct evidence of morphology and crystal orientation; however, their spatial limitations compromise the reliability and comparability of results across different studies [81]. Future research should incorporate advanced techniques with a wider field of view, such as computed tomography, confocal laser scanning microscopy, X‐ray polarography, and electron backscatter diffraction to comprehensively evaluate orientation characteristics. Additionally, it is strongly recommended that multiple sets of parallel experimental results should be reported and that statistical analyses need to be conducted, which will gradually contribute to the establishment of a widely accepted evaluation system.

### 3.3 Expanding the Focus Beyond the Conventional Zn(002) Plane

Current research predominantly focuses on achieving highly oriented Zn(002) deposition, primarily due to its flat atomic arrangement and low hydrogen evolution reaction activity. However, increasing evidence suggests that other crystal planes, such as Zn(100), Zn(101), Zn(103), and Zn(112), also yield dense and flat surfaces conducive to the long cycle life of batteries. Therefore, future investigations should transcend Zn(002) orientation deposition and explore these emerging alternative orientations, evaluating their electrochemical performance under varying current densities, areal capacities, and electrolyte environments to identify optimal deposition pathways beyond Zn(002) paradigm.

### 3.4 Correlating Orientation Control with Full‐Cell Electrochemical Performance

Although current approaches effectively guide oriented deposition and suppress dendrite growth and side reactions, they may compromise overall battery performance. For instance, introducing additives into the electrolyte can hinder ion transport, increase concentration and ohmic polarizations, and reduce the energy efficiency of full batteries. Similarly, achieving Zn(002) orientation may slow deposition kinetics, which may sacrifice the rate performance and energy efficiency of full batteries. Future research should adopt a system‐level approach to develop strategies that can ‘kill two birds with one stone’, or integrate orientation control with other approaches to enhance the overall performance of the full cell.

### 3.5 Designing Deposition Strategies Resilient to Diverse Operating Conditions

In real‐world applications, RAZBs are required to operate stably under varying rates, different charge/discharge depths, and dynamically changing temperatures. Currently, most research validates oriented deposition only at specific current densities, areal capacities, or within particular electrolyte systems, which does not adequately reflect reliability under practical conditions. Future work should prioritize the development of regulation strategies that maintain the orientation of Zn deposition and morphological stability across a broad spectrum of operating conditions, thereby bridging the gap between laboratory research and commercial deployment.

### 3.6 Developing Scalable, Application‐Driven Deposition Techniques

Current methods for regulating oriented Zn deposition are largely confined to lab‐scale demonstrations, often constrained by cost, complexity, or environmental sustainability. To facilitate practical implementation, it is imperative to develop scalable, cost‐effective, and environmentally sustainable strategies to regulate highly textured Zn deposition, while validating their efficacy in pouch cells [82, 83]. Promising directions include optimizing electrodeposition techniques to fabricate Zn electrodes and refining commercial Zn foils via heat treatment or mechanical processing. Systematically evaluating the energy consumption, cost, and environmental impact of these processing routes is crucial prior to industrial adoption.

## Author Contributions

**Ang Li**: conceptualization (lead), writing – original draft (lead), writing – review and editing (equal). **Xinyu Zhang**: conceptualization (supporting), writing – review and editing (supporting). **Maochun Wu**: conceptualization (equal), funding acquisition (lead), project administration (lead), supervision (lead), writing – review and editing (lead).

## Funding

This study was supported by the grants from the Research Grants Council of the Hong Kong Special Administrative Region, China (16205822), PolyU Start‐up Fund (1‐BDC4).

## Conflicts of Interest

The authors declare no conflicts of interest.
